# Circulating Cell-Free DNA and Colorectal Cancer: A Systematic Review

**DOI:** 10.3390/ijms19113356

**Published:** 2018-10-26

**Authors:** Veronika Vymetalkova, Klara Cervena, Linda Bartu, Pavel Vodicka

**Affiliations:** 1 Institute of Experimental Medicine of the Czech Academy of Sciences, Videnska 1083, 142 00 Prague, Czech Republic; klara.cervena13@seznam.cz (K.C.); lindabartu@gmail.com (L.B.); pavel.vodicka@iem.cas.cz (P.V.); 2 Institute of Biology and Medical Genetics, 1st Medical Faculty, Charles University, Albertov 4, 128 00 Prague, Czech Republic; 3 Biomedical Centre, Faculty of Medicine in Pilsen, Charles University in Prague, 323 00 Pilsen, Czech Republic

**Keywords:** cell-free DNA, colorectal cancer, liquid biopsy

## Abstract

There is a strong demand for the identification of new biomarkers in colorectal cancer (CRC) diagnosis. Among all liquid biopsy analysts, cell-free circulating DNA (cfDNA) is probably the most promising tool with respect to the identification of minimal residual diseases, assessment of treatment response and prognosis, and identification of resistance mechanisms. Circulating cell-free tumor DNA (ctDNA) maintains the same genomic signatures that are present in the matching tumor tissue allowing for the quantitative and qualitative evaluation of mutation burdens in body fluids. Thus, ctDNA-based research represents a non-invasive method for cancer detection. Among the numerous possible applications, the diagnostic, predictive, and/or prognostic utility of ctDNA in CRC has attracted intense research during the last few years. In the present review, we will describe the different aspects related to cfDNA research and evidence from studies supporting its potential use in CRC diagnoses and the improvement of therapy efficacy. We believe that ctDNA-based research should be considered as key towards the introduction of personalized medicine and patient benefits.

## 1. Introduction

The discovery of circulating acids (cell-free DNA-cfDNA, small and long non-coding RNAs) in body fluids attracted considerable attention in cancer research. The advantage of detection of cell-free DNA in plasma or serum is the potential to become a minimally invasive diagnostic and prognostic tool for colorectal cancer (CRC) patients [1]. Since early detection is a critical goal in cancer screenings, circulating nucleic acids represent a new class of biomarkers which can be used to improve the patients’ outcome.

Genetics represents a key role in predisposition to CRC and in its initiation, progression, and metastasis [2]. CRC provides a useful model for the study of other cancers—the various stages of tumor development can be observed. The well-defined sequence of events (from the aberrant crypt proliferation or hyperplasia via benign adenomas to carcinoma in situ and finally to metastatic carcinoma) testify the stepwise progression of this cancer, which often develops over many years [3]. The availability of biopsies is facilitated to determine that somatic mutations occur in several genes during the sequence of events. Apart from genetic events, smoking, diets, lifestyle, and other nongenetic factors are thought to have a strong impact on CRC risk.

Identifying predisposing germline mutations (heritable changes in the DNA occurring in the germ cells) together with acquired somatic mutations is important for the elucidation of the carcinogenic process and in the design of diagnostic, therapeutic, and preventive strategies. Rapid advances are being achieved in the understanding of the molecular genetics and epigenetics of CRC. Accumulating evidence has presented CRC as a heterogeneous and complex disease. Molecularly and clinicopathologically, CRC is a very heterogeneous disease and different genetic features have been observed between colon cancer (CC) and rectal tumors [4]. These features implicate that the two diseases require different treatment strategies. A standard treatment of locally advanced rectal cancer consists of a multi-modal treatment with neoadjuvant chemoradiotherapy (therapy before surgery) followed by a total mesorectal excision of the tumor [5]. The common treatment for CC is surgical resection. If metastases are not present, the radical surgical resection of the primary tumor takes place. Because the surgery may not eliminate all cancer cells, adjuvant chemotherapy treatment is used to exterminate any tumor cells that may have been missed (micrometastasis). The adjuvant chemotherapy (applied after surgery) is used as a prevention method of cancer reappearance and is recommended for all patients with stage III CC without contraindications after curative resection. The medication 5-Fluorouracil (5-FU) has been the first choice of the adjuvant chemotherapy drug for CC for many years [6]. Usually, it is combined with leucovorin, which makes 5-FU more effective. Eventually, the combination of drugs called FOLFIRI (5-FU, leucovorin, and irinotecan) or FOLFOX (5-FU, leucovorin, and oxaliplatin) is applied [7].

CRC, a common neoplasia, ranks as the second most common type of cancer (11.5% worldwide), with nearly one million new cases diagnosed every year. An increase in the CRC incidence has been recorded all over Europe in the past decade, being particularly severe in central European regions [8]. Despite the continuously growing incidence, mortality rates gradually decrease in developed countries, probably due to the implementation of early screening [9] or the integration of new endoscopic and molecular techniques into clinical practice. Close collaboration between clinicians and scientists has expanded our understanding of the histo-clinico-pathological and molecular stages in CRC.

The prognosis for patients suffering from CRC is heavily dependent on the stage at diagnosis: a 5-year survival rate is up to 90% for stage I, but only <15% for advanced stages [6]. Over half of the cases are diagnosed at a higher stage of disease (III and IV). Treatment usually involves the complete primary tumor resection and appropriate chemotherapy. While the treatment can reduce the risk of relapse and increase a patient’s survival, it can also cause severe side effects and impair the quality of life. The differences in therapy response are mainly caused by a patient’s individual inherited genetic susceptibility affecting the response to the medication [10]. Current approaches to choose and implement chemotherapy regimens for CRC patients are primarily determined by tumor staging and histopathological examination. Developing diagnostic, prognostic, and predictive biomarkers based on the personal genetic background would greatly aid oncologists in the selection of the optimal type of drugs for each patient to improve their clinical outcome.

Significant effort is currently being employed to discriminate patients who will benefit from chemotherapy [11]. There is no current ‘gold standard’ to differentiate responders from non-responders. The analyses are usually performed by comparing the group of patients showing a certain response (from partial to complete) with a group of patients lacking any response.

Here, we will summarize the latest findings on cfDNA and evidence from studies supporting its potential use in CRC diagnoses and improvement of therapy efficacy.

### 1.1. Liquid Biopsy

There is a clear clinical need for novel diagnostic and molecular tools in oncology. Precision medicine focuses on the understanding of the precise relationship between the genes and phenotype, and the stratification of diseases into subtypes according to their underlying biological mechanisms. The heterogeneity of cancer indicates that the practice of using tissue biopsies for treatment decision making or disease monitoring has obvious weaknesses. Repeated monitoring of the tumor genome for treatment response assessment is a prerequisite for personalized therapy; unfortunately, this is nearly impossible because serial biopsies are usually a health burden for the patient due to the invasiveness of the procedure [12]. Tumors are continuously in evolution, and even if several biopsies are obtained, these are limited both spatially and temporally. Moreover, in several patients, it is impossible to obtain biopsy samples and for sufficient material of adequate quality for genomic profiling, we need to be aware that reported failure rates range between 10–30% of cases [13]. It is important to keep in mind that almost all tumors treated with any therapy acquire resistance because of tumor heterogeneity, clonal evolution, and/or selection [14]. Usually, the treatment decision is based on the result from single tumor biopsy and many substantial lesions might by simply overlooked [12]. As stated above, in most cases, it is impossible to obtain a biopsy sample and no information about the genetic background of the developed metastasis is available. Moreover, during the evolution and progression of cancer, metastases might lose aberrations present in the primary lesion and this leads to differences between the primary tumor and the metastases.

**Figure 1:**
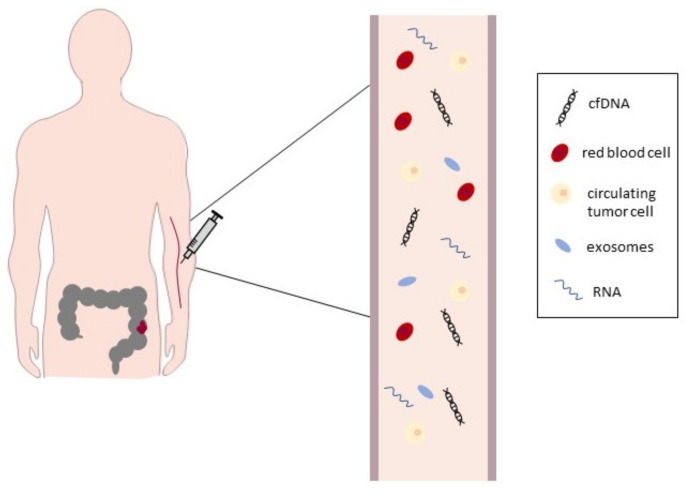
A liquid biopsy for solid tumors. A literature scan of the last few years revealed an enormous increase in liquid biopsy-based analyses [20].

With the improvements in research, it is now well known that various cells within a tumor constantly release many biomolecules such as DNA, RNA, and proteins into body fluids. These biomolecules can be either cell-free or bound to proteins or lipids, or capsulated in extracellular vesicles. Because circulating cell-free DNA (cfDNA) is a surrogate for the tumor genome, it is often referred to as a liquid biopsy [12]. Biomarkers in body fluids are thus of tremendous potential in translational research. A liquid biopsy minimizes invasive approaches to sampling cancer cells and their molecular signatures. Moreover, a liquid biopsy is a representative sample of the entire tumor, which overcomes tumor heterogeneity which is the difficult to be captured by tissue biopsy ([15], Figure 1) and represents a reliable source of diagnostic DNA and could thus replace the use of tumor tissue in diagnostic settings [16]. The fecal occult blood testing (FOBT) and colonoscopy are routinely used for the early detection of CRC. However, the sensitivity for detecting adenomas with FOBT is very low [17]. Moreover, the compliance for colonoscopy is quite low because it is time consuming, unpleasant, painful, and involving some risk [18]. A CRC screening test that accurately detects advanced adenomas with a high potential of malignant progression is needed [19]. Recently, the multitarget stool DNA test or the blood-based *Septin 9* DNA methylation test have been launched in clinical practice. A liquid biopsy analysis may also exhibit a great diagnostic potential in CRC for monitoring resistance development to treatment. These new diagnostic tools and the definition of molecular biomarkers in CRC will improve early detection and targeted therapy of CRC. We believe that the combination of FOBT and a liquid biopsy analysis may improve the performance of CRC screenings.

The main differences between the conventional analysis of tumor tissue and a liquid biopsy are the fact that tumor tissue is very genetically heterogeneous, and tumor cells are, therefore, not all genetically identical to one another. The DNA, mRNA, or microRNA profiling of tumor tissue only provides the analysis of the predominant tumor cells. Modern high-throughput techniques enable the identification of several predominant tumor cells but not of all tumor cells unless every tumor cell is analyzed. Owing to this, several tumor cells may not have been captured and tested. However, this can be omitted by analyzing the liquid biopsy, albeit with a lower tumor fraction in the plasma, due to which the sensitivity of ctDNA detection decreases. Nevertheless, tissue and liquid biopsies investigate different parameters and, therefore, do not deliver identical results.

A ctDNA/cfDNA analysis has their own advantages and disadvantages and their results can supplement each other (Table 1).

### 1.2. Cell-Free DNA

Circulating tumor DNA (ctDNA) is fragmented DNA derived from a tumor into the bloodstream and is not associated with cells. Very often the ctDNA term is confused with cfDNA, which characterizes DNA freely circulating in the bloodstream and is not necessarily of tumor origin. Because ctDNA reflects the entire tumor genome, it has earned an attraction for its potential clinical utility as a ‘liquid biopsies’. The fraction of ctDNA can range between 0.01% to 90% [21,22,23].

For the first time, cfDNA was discovered in the blood of healthy individuals by Mandel and Métais [24]. Higher cfDNA levels were first reported in the serum of patients with various types of cancer by Leon and Shapiro in 1977 and thus proved the diagnostic features of cfDNA in cancer patients. Similarly, metastatic cancer patients had a higher level of cfDNA than non-metastatic patients. The authors also observed that after radiation therapy, cfDNA levels decreased; albeit with constant or increasing levels of cfDNA being associated with a worse prognosis or relapse. Leon et al. hypothesized that cfDNA in serum could represent a promising biomarker for evaluating therapy response [25]. The following studies aimed to prove that cfDNA characterizes neoplastic features of the tumor. In 1989, Stroun et al. [26] suggested that part of the cfDNA in the plasma of cancer patients originates from tumor cells. Tumor-specific aberrations, like mutations in oncogenes and tumor suppressor genes [27], microsatellite instability (MSI) [28], and DNA methylation [29] were identified in cfDNA and studies confirmed the release of cfDNA into the circulation by tumors.

Recent reports have shown the possibility of reconstructing the genome of tumors from ctDNA as ctDNA represents a potential surrogate of the entire genome [30,31]. This means that genetic and epigenetic signatures in ctDNA correspond to those in the primary tumor and may reveal the tumor-specific (epi)-genetic spectrum [32]. This implies ctDNA is directly released from tumors. Several health conditions may be associated with increasing levels of cfDNA such as inflammation, tissue trauma, autoimmune diseases, or cancer [33,34]. Concerning cancer and cfDNA studies, most of the studies confirmed that patients with cancer have higher levels of cfDNA than patients with benign diseases or healthy individuals [35,36,37,38].

cfDNA could be found in serum, plasma, and other body fluids like urine or saliva [39,40] but the mechanisms of release into the bloodstream are not completely understood. In general, cfDNA may be derived from a primary tumor, metastatic lesions, or circulating tumor cells (CTCs) [41]. There are two ways of release—passive and active. Passive release means by necrotic and apoptotic cells and active secretion can be mediated by nucleated cells such as lymphocytes. Regardless, the major source of cfDNA in plasma is from the apoptotic or necrotic cells [42]. A few authors suggest that malignant tumors exhibit a higher degree of necrosis which corresponds to an increase in ctDNA levels. Diehl et al. [43] suggested that DNA fragments in the circulation are derived from necrotic tumor cells absorbed by macrophages. However, Leon et al. [25] showed that radiation therapy in patients induces cell necrosis and observed a 90% decrease in cfDNA levels. In addition, several studies stated that cfDNA is derived from active cellular secretion, for example, macrovesicles such as exosomes [44,45]. Recently, it was hypothesized that all cells that are alive actively release DNA into the circulation [12]. Therefore, both apoptosis and necrosis, alongside with active secretion play important role in the cfDNA presence in liquid biopsies.

cfDNA is typically fragmented into 180 bp fragments, corresponding to the length of DNA wrapped around a nucleosome and protected from degradation [12,46]. Another peak representing the multimers of nucleosomes in a minor fraction of the total can be observed [12]. A peak at 166 bp [47,48] corresponds to the length of DNA wrapped around a nucleosome (~147 bp) with DNA associated histone H1. Depended on the size of the fragment of cfDNA, the source of release can be identified. cfDNA fragments produced by apoptotic cells are about 200 bp long and contrarily cfDNA released from necrotic cells has a variable shorter length [42] and may thus increase the proportion of shorter fragments and lead to low integrity [49]. The ratio of longer to shorter DNA fragments is named the cfDNA integrity number and is determined as a ratio of two qPCR products of different lengths. Umetani et al. developed a method for measuring the integrity of cfDNA by qPCR for ALU repeats (247 bp ALU vs. 115 bp ALU) [50]. The reason for choosing these sequences is the argument that ALU repeats are the most abundant repeated sequences in the human genome [51]. ctDNA molecules are also shorter than non-mutated cfDNA molecules in plasma [48,52,53,54,55]. Longer cfDNA fragments (>1000 bp) were observed in healthy volunteers [56]. The authors hypothesized that the release into circulation in healthy individuals may be associated with exosomes [44,56,57,58] and in cancer patients by necrosis from tumor cells [42].

The average concentration of cfDNA in the blood serum of healthy volunteers was 13 ng/mL, whereas in cancer patients the mean level was 180 ng/mL [25]. The amount of circulating DNA, whether tumoral or not, is limiting: a range between 1 to 10 ng per ml plasma [59,60], which correspond to 1500 to 3000 copies of the haploid genome [61]. A substantial variation in ctDNA levels may arise from an interindividual difference. Poor tumor vascularization could hamper ctDNA release into the bloodstream or, on the other hand, could support ctDNA release by producing hypoxia and cell death [58]. When the plasma cfDNA and serum cfDNA concentration was compared, it was found out that the serum concentration is 3–24 higher than in plasma [62,63]. Jung et al. confirmed these differences and related it to the time delay and storage temperature of blood before centrifugation. Higher levels of cfDNA in the serum can also be caused by contamination by cells during the clotting process. For this and other reasons for the analysis of tumor-specific DNA, it is recommended to use the plasma as a source of cfDNA due to lower concentrations of awild-type DNA background [64]. Many researchers choose plasma for their analysis because it is less likely to be contaminated by leucocytes than serum [65]. It is important to remember that most of cfDNA originate from normal cells, mostly from the hematopoietic compartment, and ctDNA generally represents only a minor fraction of it, possibly 0.1% of the total amount, or even less [66,67].

cfDNA is considered a good biomarker and can be characterized in disease monitoring by two options—quantitative and/or qualitative changes. Quantitative changes include differences in the concentration of cfDNA and qualitative changes are represented by gene mutations, loss of heterozygosity, DNA copy number variations, methylation, microsatellite instability (MSI), etc.

As ctDNA represents only a very small proportion of cfDNA, very sensitive and reliable detection methods are required. Detection methods can be divided into two groups: (i) the targeted approach that allows for the detection of specific alterations, and (ii) the untargeted approach that allows for the identification of events a priori, for example, whole exome or genome sequencing. Levels of cfDNA/ctDNA are measured mainly by real-time PCR (RT-PCR) [68]. Digital PCR (dPCR), RT-PCR [27,69] or sequencing methods [70], beads Emulsion Amplification and Magnetics (BEAMing) are used for the detection of point mutations. Sequencing methods can identify structural rearrangements, chromosomal copy-number changes, and structural alternations of ctDNA in circulation [71,72]. Different cfDNA/ctDNA detection techniques with their pros and cons are summarized in Table 2.

### 1.3. Preanalytical Considerations

The preparation of cfDNA for analysis is easy to implement. For the isolation of cfDNA, about 5–10 mL of blood collected in tubes with anticoagulants is required. CfDNA could be processed from serum as well but with a lower priority due to the lysed cellular DNA that may have an impact on relative levels of cfDNA. In the blood, cfDNA has a limited stability because of the DNase activity and therefore the preparation of cfDNA should not overstep three hours after blood collection [41]. Cell lysis needs to be avoided in order to prevent the release of a large amount of non-mutant DNA, potentially leading to a false negative result [67].

The lack of consistency between several protocols for sample handling and methodologies used for cfDNA analyses represent the major obstacles in translating cfDNA based research into clinical practice. Therefore, Messaoudi et al. [75] and recently Nikolaev [67] determined the optimal preanalytical protocols for cfDNA analyses:

- Plasma is a better source of cfDNA than serum since it avoids blood cell genomic DNA contamination
- EDTA or cell-free DNA^TM^ collection tubes prevent blood cell lysis by keeping tubes at 4 °C
- Blood must be processed within a maximum of 4 h following blood drawing to preserve cfDNA concentration and fragmentation
- To ensure any absence of cells in plasma, first, centrifugation is recommended at 1200–1600 g for 10 min and, second, microcentrifugation at 16,000 g for 10 min, (the second step can be indifferently realized before or even after storage of plasma samples)
- Plasma samples must be stored at −80 °C for up to nine months (samples are sensitive to freeze-thaw cycles)
- cfDNA extracts may tolerate a maximum of three freeze-thaw cycles and storage at −20 °C for up to three months

## 2. Results

### 2.1. Cell-Free DNA as a Physiological Mobile Genetic Element

Mobile genetic elements play an important role in shaping biotic genomes and bringing about evolutionary transformations [76]. Recently it was observed that cfDNA, after entering healthy cells, integrated into their genomes and led to DNA damage [77].

Genomic integration of cfDNA might then lead to DNA rearrangements, translocations, and deletions [78] and ultimately induce the aging of cells [79] or may activate chemoresistance.

A process called genometastasis, where the cfDNA in the plasma may participate in tumorigenesis and the development of metastases via transfection-like uptake of nucleic acids by susceptible cells was recently introduced. García-Olmo [80] cultured NIH-3T3 mouse cells with plasma samples from CRC patients bearing *KRAS* mutation (codon 12 in exon 1), as well as with plasma from healthy individuals. The authors detected mutated human *KRAS* sequences in cultures of NIH-3T3 cells after the start of incubation and these sequences were still detectable at the end of the experiment, even ~3 weeks after the removal of human plasma from the culture medium. NIH-3T3 cells that were cultured with plasma from healthy individuals were negative. NIH-3T3 cells (p16-deficient cells) were able to stably incorporate foreign DNA during a simple incubation with plasma from cancer patients. Another study demonstrated the integration of ctDNA into nuclear DNA of leukemic cells and suggested that this process may occur through non-homologous end-joining [81]. This all can conclude that cfDNA, far from being an inert molecule, has some biological functions of their own that are deleterious to healthy cells of the body.

### 2.2. Cell-Free DNA and Colorectal Cancer

**Figure 2:**
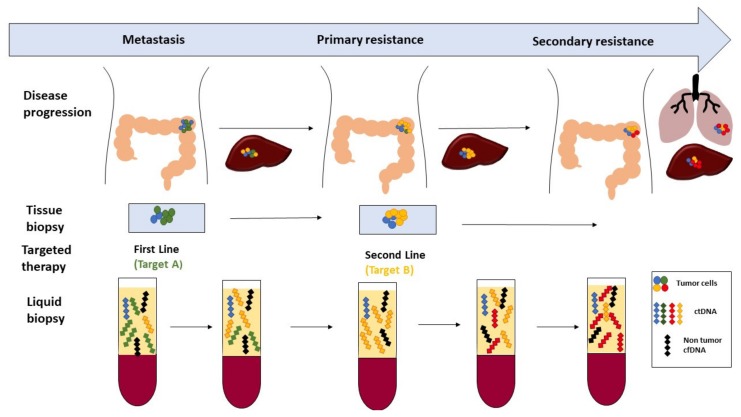
A liquid biopsy to monitor the therapy response and resistance. A hypothetical scenario of the developing chemotherapy resistance of colorectal cancer patients: first-line therapy is based on the primary tumor and relevant changes in the metastasis might be overlooked, therefore, leading to primary resistance. After switching to second-line therapy, secondary resistance may arise. Genetic changes of the resistant clones can be monitor using liquid biopsy and, therefore, the resistance mechanism might be recognized before disease progression (adapted from Heitzer et al. [12]).

Analysis of ctDNA is a promising new tool in oncology. ctDNA mutational content can provide invaluable information on the genetic background of a tumor, and assist oncologist in deciding on therapy, or in following the residual disease (Figure 2, Table 3).

#### 2.2.1. Cell-Free DNA as a Diagnostic Biomarker in Colorectal Cancer

The average concentration of serum cfDNA from CRC patients was 5-times higher than that in the serum of healthy controls, while in plasma, it was 25–50 times higher in CRC patients than in the plasma of healthy controls [91,92,95,126]. Several authors analyzed the cfDNA concentration differences in plasma between rectal and colon cancer patients. Colon cancer patients evinced higher cfDNA concentration than patients with rectal cancer (RC) (colon: 500 ng/mL, rectum: 250 ng/mL in plasma) [98], while Cassinotti et al. observed a higher concentration of cfDNA in patients with RC [110].

Activating mutations in the *KRAS* gene are predictors of poor response in patients with metastatic CRC receiving anti-EGFR (epidermal growth factor receptor) antibody-based therapy. Therefore, these patients are routinely tested for the presence of *KRAS* mutations before receiving biological therapy [160,161]. Numerous studies tried to prove that KRAS mutation can be detected in cfDNA and may serve as a diagnostic tool. Already in 1997, Anker at al. [82] postulated that the genetic analysis of plasma DNA may have clinical applications in the future. In their study, *KRAS* abnormalities were analyzed in 14 tumors and plasma from CRC patients. Similarly, de Kok and Kopreski, in 1997 [83,84], analyzed the serum of CRC patients for *KRAS* mutations. Back in 1992, Sidransky et al. [162] reported that *KRAS* mutations in tumors from CRC patients and stool samples were identical. The possibility of detecting mutations in body fluids like plasma and serum was analyzed in many other studies [86,115,126]. Wang et al. detected *KRAS* mutations in tumor tissue and evaluated the presence of these mutations in serum samples. Their results showed that about 45% of CRC patients with *KRAS* mutations in tumor tissues evinced these mutations in cfDNA. Interestingly, *KRAS* mutations were not detected in the cfDNA of the healthy control group [27]. The concordance between *BRAF* and *KRAS* mutation analysis in tumor tissue and matched plasma was analyzed further. The *KRAS* mutation analysis showed a 96% concordance with tumor tissue and plasma cfDNA while the *BRAF* mutation analysis showed a 100% concordance [23]. Very recently, Sclafani et al. [142] analyzed the most frequently mutated *KRAS* hotspot mutation (i.e., G12D, G12V, and G13D) in rectal cancer tissue and corresponding plasma and did not observe much of a difference in the rate of *KRAS* mutation in plasma between patients with wild-type *KRAS* and *KRAS*-mutated tumors. Interestingly, by analyzing of plasma ctDNA the presence of additional *KRAS* mutations that were not detected on tissue were identified. Bettegowda et al. [21] detected that the sensitivity of ctDNA for the detection of clinically relevant *KRAS* gene mutations was 87% for stage IV CRC patients while in stage I CRC patients, a decrease to 47% was observed. Yang et al. [152] monitored that the ctDNA concentration increased with tumor size and cancer stage, where Stage IV patients evinced higher ctDNA levels than Stage I patients. Surprisingly, the Stage IV CRC patients showed only slightly over one mutated gene per patient than those with CRC Stage I.

Epigenetic analysis of cfDNA might contribute to the identification of gene hypermethylation [86] or the type of cell that leads to a rise of cfDNA fragments [163], and thus provide information about the tumor microenvironment which usually lacks somatic mutations. The methylation status of cfDNA was studied less intensively [103,122].

Recently, Li et al. aimed to prove the possibility of detecting copy-number variation (CNV) in the serum and in plasma of CRC patients, individuals with polyps and healthy volunteers. The analysis showed more prominent copy number changes in plasma than in serum. From 80 patients with CRC, CNV was detected in 39 of them and the majority of them were patients with an advanced stage. The most common changes included whole chromosomes gains on chromosomes 2, 7, 13, and 20; partial gains at chromosomes 8, 12, 13, and 20; and partial losses at chromosomes 1, 3, 4, 8, 17, 18, and 22 [135].

#### 2.2.2. Cell-Free DNA as a Predictive Biomarker in Colorectal Cancer

Proper information on the clinical/pathological staging and the possibility of identifying cancer patients with a high likelihood of recurrence, or risk of clinical toxicity, are vital for the development of more efficient/less toxic treatment strategies.

The treatment of CRC has advanced over the past several years with the introduction of several active agents. Determining which patients to treat with chemotherapy and choosing the optimal treatment regime would allow oncologists to maximize the benefit of chemotherapy. Several prognostic and predictive markers have been identified and include oncogenes, tumor suppressor genes, genes involved in angiogenic and apoptotic pathways and cell proliferation, and those encoding targets of chemotherapy.

Zitt et al. [101] evaluated whether the concentration of cfDNA has the potential to serve as a marker for therapy monitoring during the treatment course of locally advanced rectal cancer patients. As the standard treatment for locally advanced RC, preoperative chemoradiotherapy (CRT) is considered. The changes in cfDNA concentration might be also observed before, after, and at the end of the preoperative CRT. The authors divided their population into responders (T0–T2 stage) and non-responders (T3–T4 stage). Both groups evinced a similar median plasma cfDNA concentration before and after the end of CRT. At the end of the treatment, the responders exhibited a decrease in cfDNA, whereas, in non-responders, the cfDNA increased. This is in agreement with other cancer research stories, in which the authors demonstrated that the response is associated with a decrease in the plasma cfDNA level, whereas no change or even an increase in the amount of cfDNA was seen in patients who did not respond to the therapy [25,164,165].

In a recent report on four locally advanced rectal cancer patients whose ctDNA was tracked in serial blood samples by using two patient-specific chromosomal rearrangements, Carpinetti et al. [121] showed an overall lack of correlation between the normalization of ctDNA and the amount of the residual disease in the surgical specimens after neoadjuvant CRT. However, changes in ctDNA levels after surgery appeared to predict the tumor recurrence.

In 2002, Diaz et al. [105] studied whether mutant *KRAS* ctDNA could be detected in the serum of CRC patients receiving monotherapy with panitumumab, a therapeutic anti-EGFR antibody. Authors found that 9 out of 24 patients whose tumors were initially without *KRAS* mutations developed detectable mutations in the *KRAS* gene in their sera. This suggests that *KRAS* mutations act as a mediator in acquired resistance to EGFR blockade and that these mutations can be detected in a non-invasive manner. Mohan et al. [115] reported the acquirement of resistance to anti-EGFR therapy with developed *KRAS* mutations and that resistant clones in the bloodstream had been detectable several months before the progression clinically manifested. A similar was we observed in the studies of Valtorta et al. [166], Misale et al. [167], and Bardelli et al. [168]. Another study focused on the abundance of mutant *KRAS*/*BRAF* alleles in the plasma of metastatic CRC patients at the baseline and before each cycle of third-line treatment with cetuximab and irinotecan [117]. cfDNA and *KRAS* levels decreased from the baseline to cycle 3 and increased at the time of progression. The decrease was larger in responding patients than in non-responding patients. The same group also investigated the total cfDNA in CRC patients during treatment with second-line chemotherapy and cfDNA in healthy controls [118]. cfDNA levels were significantly higher in CRC compared to controls. Patients with high cfDNA levels had a shorter survival after irinotecan-based therapy compared to those with lower levels.

The cfDNA integrity number (i.e., a ratio between long and short DNA fragments) turned out as an independent marker on CRT. The cfDNA integrity index was lower in responders compared to non-responders after CRT (as defined by the degree of tumor regression according to the Mandard score) only after completion of fluoropyrimidine-based CRT and, generally, patients with CRC had a 10-times higher cfDNA integrity number than in healthy subjects [104]. The DNA integrity index was observed to be the independent predictive factor of response to neoadjuvant treatment in multivariate analysis. Sun et al. [113,169] confirmed the potential of the DNA integrity index as they observed an association between this parameter (both at the baseline and after neoadjuvant treatment) and tumor regression grading according to the Dworak’s score in rectal cancer patients who received an oxaliplatin-based CRT for cT3-4 and/or N+ rectal tumors. The authors also observed that the plasma level of the *KRAS* mutation (codon 12) decreased with CRT in all patients with no difference between responders and non-responders and that the higher *MGMT* promoter methylation status at baseline DNA was associated with a better tumor response.

#### 2.2.3. Cell-Free DNA as a Prognostic Biomarker in Colorectal Cancer

The prognosis of patients with CRC is highly impacted by various factors at the time of diagnosis (tumor localization, quality of surgical procedures, gender, age, and the patient’s overall performance status).

Recently, the prognostic and/or predictive values of ctDNA in CRC have attracted the most intense interest. It is hypothesized that the mutational pattern observed in ctDNA might aid to stratify patients’ group into molecular subtypes with different prognoses, for example in diffuse large B lymphoma [169] or breast cancer [170]. Yi et al. [170] observed that the number of somatic mutations increased after therapy and the fractions of truck mutations were positively associated with targeted therapy. Studies uncovered that presence of ctDNA could be a reliable prognostic factor correlated with poorer outcome and support the idea of ctDNA as a non-invasive biomarker of minimal residual disease (Table 3.). Cassinotti et al. [110] and Frattini et al. [92] observed that after primary resection, the concentration of cfDNA significantly decreased, however, in patients with a relapse, the cfDNA levels dramatically increased, while, in “disease-free” patients, the cfDNA concentration still had a decreasing tendency. The preoperative measurement of cfDNA might contribute to the better estimation of prognosis and a post-operative measurement could represent a promising tool for the early detection of recurrence. A higher cfDNA concentration significantly correlated with a worse survival in other studies [100,126]. Diehl et al. [97] observed that CRC patients with detectable ctDNA after surgery generally relapsed within 1 year and considered ctDNA as a highly specific biomarker of tumor dynamics. This might indicate that ctDNA levels in plasma after surgery represent a reliable marker of residual disease. The utility of serial samplings proved the assumption of relapse prediction. Reinert et al. [123] observed a 2–15 (mean 10) months lead time on detection of metastatic recurrence compared to conventional follow-up. El Messaoudi et al. [129] postulated that all cfDNA parameters might be considered as prognostic markers: patients with higher levels of mutant ctDNA and higher mutation loads for the detected mutations in *KRAS* or *BRAF* genes evinced shorter OS. Similarly, Xu et al. [120] observed that patients with *KRAS* mutations evinced worse overall survival (OS) than *KRAS* wild-type patients. Higher ctDNA levels were associated with a higher risk of recurrence and a worse OS in CRC patients treated with surgery, chemotherapy, radiotherapy, or targeted therapy [87,112].

The detection of ctDNA in CRC patients with stage I, II, or III was associated with a shorter survival and disease recurrence. Lecomte et al. [86] observed a decreased 2-year survival in patients with cfDNA in plasma when compared to those without cfDNA in their plasma and suggested that patients with a high risk of recurrence can be distinguished by the analysis of tumor-derived ctDNA. Wang et al. [27] analyzed the presence of mutations in *APC*, *KRAS*, and *TP53* genes in the serum of CRC patients and observed a significant correlation between the ctDNA and developed postoperative recurrence. Similarly, Tie et al. [130] in stage II CRC patients noticed that ctDNA detected postoperatively increased when not treated with chemotherapy. On the other hand, patients with negative ctDNA postoperatively were at a lower risk of radiological recurrence. Similarly, authors observed that ctDNA measurements seem to be a more sensitive marker of recurrence prediction than CEA levels. More than 80% of CRC patients were ctDNA positive at the time of recurrence, while only 41% of CRC patients evinced elevated CEA levels. Lately, Reinert et al. [123] and Carpinetti et al. [121] found that ctDNA monitoring in CRC patients may render an earlier cancer recurrence and therapy response in comparison with standard CEA measurements or radiological diagnosis. Similarly, Yang et al. [152] detected elevated CEA levels in 20% of Stage I CRC patients in comparison to 86% of Stage IV patients, while ctDNA levels above the limit 0.01 ng/µL were detected in 96% of all CRC patients (including Stage I). This hallmark points to the idea that ctDNA as a supplement to traditional diagnostics with real-time tumor information and shows a much greater sensitivity than other tumor biomarkers.

The presence of methylation of *HLTF* and *HPP1* genes was associated with a worse survival [94,107]. Lee et al. [111] analyzed the promoter methylation of the Septin 9 gene among CRC patients’ stage I-II and suggested that the methylation of the Septin 9 might be associated with lower disease-free survival. Recently, Herbst et al. [133] suggested that the detection of *HPP1* methylation in cfDNA might be used as a prognostic marker and an early marker to identify patients who will likely benefit from a combination of chemotherapy and bevacizumab.

In summary, Fan et al. [171], in their comprehensive review, revealed that a ctDNA-positive status is associated with a worse prognosis. The authors performed a systematic review of data from published studies until December 2016.

### 2.3. Cell-Free DNA in Other Body Fluids

The liquid biopsy research is continuously developing. Many researchers are looking for new non-invasive approaches to detect and analyze ctDNA in other body fluids. Fujii et al. [172] monitored 56 patients with CRC *KRAS* mutations in their urine, plasma, and archival tumor tissue using mutation enrichment PCR coupled with NGS [172]. The concordance between ctDNA in urine and mutant *KRAS* in the tumor was 89% (sensitivity 80%, specificity 100%). In their study, patients had significantly fewer mutant cfDNA *KRAS* copies in urine during systemic therapy than at baseline or disease progression. Compared with no changes or increases in urine mutant cfDNA *KRAS* copies during therapy, decreases in these measures were associated with a longer median time to treatment failure. These preliminary findings suggest that ctDNA in urine may also reflect tumor dynamics and serve as a valid method of monitoring during treatment.

Several authors focused their study on the identification of stool biomarkers in CRC research. Ahlquist et al. [173] examined the possibility of cfDNA alterations in stools to discriminate between subjects with CRC and healthy individuals. *APC*, *TP53*, and *KRAS* gene mutations and BAT26 microsatellite instability were evaluated and the authors reported a 91% sensitivity and 93% specificity in detecting CRC. The same authors, Ahlquist et al. [174], used a next-generation stool DNA test based on a quantitative allele-specific real-time target and signal amplification assay with the aim of detecting early-stage CRC. The sensitivity of 85% for CRC and a specificity of 89% were obtained.

## 3. Conclusions

The aim of this review was to describe the different aspects related to cfDNA/ctDNA and evidence from studies supporting its potential use in CRC diagnoses and the improvement of therapy efficacy and prognosis. Most of the articles found that the presence of ctDNA in serum or plasma is associated with a worse survival possibility for patients with CRC.

The potential of the liquid biopsy in CRC research is attracting many scientists and has started to be a part of several clinical trials. However, before implementation of the liquid biopsy in clinical practice, it is necessary to set up standardized pre-analytical methodologies, including blood collection, processing, and storage and DNA extraction, quantification and, of course, validation in large prospective clinical studies [12]. Similarly, a better understanding of the origin and biology of cfDNA and ctDNA would help in the implementation of the results [175]. The impact of apoptosis and necrosis and the active release should be also explored. Our limited understanding of the release and clearance mechanisms of cfDNA hinders the interpretation of the current research [58].

Despite the ctDNA analysis having a great sensitivity and specificity compared with conventional diagnostic tools, taking a multi-marker approach may offer a more comprehensive insight into patient prognoses or therapy responses [23,59,129].

Since ctDNA provides real-time molecular information to track treatment response and relapse, it has a big potential to unravel drug-resistance mechanisms. With the advantage of next-generation sequencing, multiple mutations in ctDNA could be identified. Precision medicine may change the clinical practice by adapting treatment choices based on an individual’s genetic background.

In the future, clinical trials on the ctDNA-based decision made in therapy selection would be definitive. Several trials of the clinical utility of ctDNA analyses for treatment monitoring are now being carrying out on NSCLC and breast cancer patients [176,177]. In summary, this emphasizes the fact that ctDNA based research is translating from exploratory research towards clinical trials in which ctDNA is acting as a decision-making tool.

These all support the further development of ctDNA as a biomarker for the detection of early diseases and minimal residual diseases following curative resection, relapse assessment, treatment response, and the development of chemoresistance. As a summary, we can conclude that the liquid biopsy should be considered as key towards the introduction of personalized medicine and patient benefits.

## 4. Methods

We searched the Ovid MEDLINE and PubMed databases using cell-free DNA, circulating tumor DNA, liquid biopsy and colorectal, colon and rectal cancer as keywords without any exclusion. We also searched bibliographies manually.

## Figures and Tables

**Table 1:** The advantages and disadvantages of a liquid biopsy.

Advantages	Disadvantages
Cost-effective	Lack of standard operating protocol
Non-invasive	Released by both healthy and tumor cells
Rapid	Requirement of sensitive and specific methods
Comprehensive tumor profile	False-positive and false-negative results
Minimal pain and risk	Personnel microenvironment may influence the released of cfDNA amount
Serial assessments	Not-standardized cfDNA/ctDNA concentration as a cancer biomarker
Directly able to assess for specific mutations	
Present in many biological fluids	
Potential to evaluate prognosis, recurrence, response to therapy	
Detection of minimal residual disease	
Assessment of cancer high-risk populations	
Enabling of early cancer diagnosis	
Evaluation of tumor heterogeneity	

**Table 2:** A summary of ctDNA detection techniques (adopted from References [73,74]).

Technique	Limit of Detection	Type of Alteration Detection	Advantages	Disadvantages
PCR based approaches (COLD-PCR, PNAs-LNA, ARMS, etc.)	0.1–1%	SNV, indels	Low cost; Easy to perform	Low sensitivity; A limited number of studied genes at a time; Genes need to be pre-determined
Digital PCR (ddPCR and BEAMing)	0.05% or less	SNV, indels, CNV	High sensitivity and specificity; Reasonable cost; Easy to perform	A limited number of studied genes at a time; Genes need to be pre-determined
NGS (Deep sequencing, TAM-seq, Safe-Seqs, CAPP-Seq, cSMART, digital sequencing)	0.01–2%	SNV, indels, CNV, rearrangements	Allows more genes to be analyzed at a time	Wide range of sensitivity depending on the NGS platform used (PCR amplicon strategies are more sensitive and less expensive than whole genome or exome sequencing); Higher cost
SERS-nanotags	0.01%	SNV	Reduced susceptibility to photobleaching; Bandwidths are significantly narrower	Raman signal deterioration upon prolonged laser illumination
UltraSEEK	0.01%	SNV, indels	Low cost and low DNA input	Lower sensitivity

ctDNA: circulating tumor DNA, COLD-PCR: co-amplification at lower denaturation temperature; PNAs-LNA: peptide nucleic acid-locked nucleic acid; ARMs: amplification refractory mutations system; ddPCR: droplet digital PCR; BEAMing: bead emulsion amplification and magnetics; NGS: next-generation sequencing; TAM-Seq: tagged-amplicon deep sequencing; Safe-Seq: safe sequencing system; CAPP-Seq: cancer personalized profiling by deep sequencing; cSMART: circulating single molecule amplification and re-sequencing technology; SERS: surface-enhanced Raman spectroscopy; UltraSEEK: high-throughput multiplex ultrasensitive mutation detection; SNV: single nucleotide variation: CNV: copy number variation.

**Table 3:** An overview of the studies investigating cfDNA/ctDNA in relation to CRC diagnosis, therapy outcome, and prognosis.

References	Patients	Controls	Origin of the Study	Source of cfDNA/ctDNA	Abnormalities	Methodology	Target	Clinical Relevance
Leon et al. [25]	9	55	USA	Serum	Concentration			Diagnostic
Anker et al. [82]	14		Switzerland	Plasma	Mutation	PCR	*KRAS*	Diagnostic
de Kok et al. [83]	14	-	Netherland	Serum	Mutation		*KRAS*	Diagnostic
Kopreski et al. [84]	31	28	USA	Plasma Serum	Mutation	PCR	*KRAS*	Diagnostic
Hibi et al. [85]	44	-	USA	Serum	Mutation	PCR	*KRAS* *TP53*	Diagnostic
Lecomte et al. [86]	58	-	France	Plasma	Mutation Methylation	MS-PCR qPCR	*KRAS* *p16*	Prognostic
Ryan et al. [87]	94	-	Ireland	Serum	Mutation	PCR sequencing	*KRAS2*	Diagnostic Prognostic
Wang et al. [27]	104	50	Taiwan	Serum	Mutation Concentration	PCR-SSCP	*APC* *KRAS* *TP53*	Diagnostic
Leung et al. [88]	49	41	Hong Kong	Serum	Methylation	MethyLight	*APC* *hMLH1* *HLTF*	Diagnostic
Lindforss et al. [89]	25	-	Sweden	Plasma	Mutation	PCR	*KRAS*	Prognostic
Bazan et al. [90]	66	-	Italy	Plasma	Mutation Methylation	PCR	*KRAS* *TP53* *p16^INK4a^*	Prognostic
Flamini et al. [91]	75	75	Italy	Serum	Concentration	qPCR		Diagnostic
Frattini et al. [92]	70	20	Italy	Plasma	Concentration	qPCR		Diagnostic Prognostic
Trevisiol et al. [93]	86		Italy	Serum	Mutation	qPCR	*KRAS2*	Diagnostic Prognostic
Wallner et al. [94]	38	20	Germany	Serum	Methylation	MS-PCR	*HPP1/TPEF* *HLTF* *hMLH1*	Prediction
Boni et al. [95]	67	67	Italy	Plasma	Concentration	qPCR		Diagnostic
Nakayama et al. [96]	94	-	Japan	Serum	Methylation	MS-PCR	*p16*	Diagnostic
Diehl et al. [97]	18	-	USA	Plasma	Concentration	qPCR		Diagnostic Prognostic
Frattini et al. [98]	70	20	Italy	Plasma	Concentration Mutation Methylation	qPCR MS-PCR ME-PCR	*KRAS* *p16^INK4a^*	Diagnostic Predictive
Lofton-Day et al. [99]	133	179	Germany	Plasma	Methylation	MS-PCR	*TMEFF2* *NGFR* *SEPT9*	Diagnostic
Schwarzenbach et al. [100]	55	20	Germany	Serum	Concentration	qPCR		Diagnostic
Su et al. [39]	20	-	USA	Serum Plasma Urine	Concentration Mutation	PCR	*KRAS*	Diagnostic
Zitt et al. [101]	26	-	Austria	Plasma	Concentration	qPCR		Prognostic Predictive
DeVos et al. [102]	97	172	Germany	Plasma	Methylation	MS-PCR	*SEPT9*	Diagnostic
Herbst et al. [103]	106	-	Germany	Serum	Methylation	MethyLight	*HLTF HPP1/TPEF*	Prognostic
Agostini et al. [104]	67	35	Italy	Plasma	Concentration Dna Integrity	qPCR		Predictive
Herbst et al. [103]	106	-	Germany	Plasma	Methylation	MS-PCR	*HPP1/TPEF* *HLTF* *NEUROG1*	Diagnostic
Diaz et al. [105]	28	-	USA	Serum	Mutation	PCR	*KRAS*	Diagnostic Predictive
Morgan et al. [106]	71	-	UK	Plasma Serum	Mutation	qPCR	*KRAS*	Diagnostic
Phillip et al. [107]	311	-	Germany	Serum	Methylation	MS-PCR	*HLTF HPP1*	Prognostic
Spindler et al. [108]	108	-	Denmark	Plasma	Concentration Mutation	qPCR	*KRAS*	Prognostic Predictive
Bai et al. [109]	106	-	China	Plasma	Mutation	PCR	*KRAS*	Diagnostic Prognostic
Cassinotti et al. [110]	223	-	Italy	Plasma	Concentration	qPCR		Prognostic
Lee et al. [111]	101	96	Korea	Plasma	Methylation	PCR	*Septin9*	Diagnostic
Spindler et al. [112]	211	-	Denmark	Plasma	Mutation	qPCR	*KRAS* *BRAF*	Diagnostic Predictive Prognostic
Sun et al. [113]	34	10	China	Plasma	Concentration Methylation Mutation	qPCR MS-PCR PCR-RFLP	*MGMT* *KRAS*	Diagnostic Predictive
Bettegowda et al. [21]	24	-	USA	Plasma	Concentration Mutation	PCR	*KRAS*	Diagnosis
Kuo et al. [16]	52	-	Taiwan	Plasma	Mutation	PCR	*KRAS*	Predictive
Lin et al. [114]	133	-	Taiwan	Plasma	Mutation	qPCR	74 genes	Prognostic
Mohan et al. [115]	10	-	Austria	Plasma	Mutation	WGS	*KRAS* *BRAF PIK3CA* *EGFR*	Diagnostic Predictive
Perrone et al. [116]	170	-	Italy	Plasma	Mutation Concentration	ME-PCR, qPCR	*KRAS*	Diagnostic
Spindler et al. [117]	108	-	Denmark	Plasma	Mutation Concentration	PCR	*KRAS* *BRAF*	Predictive
Spindler et al. [118]	100	100	Denmark	Plasma	Concentration Mutation	PCR	*KRAS*	Diagnostic Predicitve
Tham et al. [119]	150	-	Singapore	Serum	Methylation	MS-PCR	*TAC1* *Septin9* *NELL1*	Prognostic
Thierry et al. [23]	106	29	France	Plasma	Mutation	qPCR	*KRAS* *BRAF*	Diagnostic Predictive
Xu et al. [120]	242	-	China	Plasma	Mutation	PCR	*KRAS*	Prognostic
Carpinetti et al. [121]	4	-	Brazil	Plasma	Chromosomal Rearrangements	SOLiD		Predictive
Lin et al. [122]	353	-	Taiwan	Plasma	Methylation	Methylation array	>450,000 CpG sites	Diagnostic
Reinert et al. [123]	118	-	Denmark	Plasma	Concentration	ddPCR		Diagnostic
Sefrioui et al. [124]	34	-	France	Plasma	Mutation Concentration	dPCR	*KRAS*	Diagnostic Prognostic
Siravegna et al. [125]	100	-	Italy	Plasma	Mutation	PCR	*KRAS*	Prognostic Predictive
Spindler et al. [126]	229	100	Denmark	Plasma	Mutation Concentration	qPCR	*KRAS*	Diagnostic Prognostic
Liu et al. [127]	165	-	Singapore	Serum	Methylation	MS-PCR	*SST*	Prognostic
Matthaios et al. [128]	155	-	Greece	Plasma	Methylation	MS-PCR	*APC* *RASSF1A*	Prognostic
El Messaoudi et al. [129]	97	-	Francie	Plasma	Mutation Concentration	qPCR	*KRAS* *BRAF*	Diagnostic Prognostic
Tie et al. [130]	230	-	Australia	Plasma	Mutation	PCR	*APC* *TP53* *KRAS*	Prognostic
Agah et al. [131]	74	-	Iran	Plasma	Concentration	qPCR		Diagnostic
Bhangu et al. [132]	30	17	Austria	Plasma	Concentration	qPCR		Diagnostic
Herbst et al. [133]	467	-	Germany	Plasma	Methylation	MS-PCR	*HPP1*	Predictive Prognostic
Kloten et al. [134]	50	8	Germany	Plasma	Concentration	qPCR mutation	*KRAS*	Diagnostic
Li et al. [135]	80	35	USA	Serum Plasma	Concentration Cnvs	WGS		Diagnostic Prognostic
Pereira et al. [136]	128	-	USA	Plasma	Mutation	sequencing		Diagnostic
Yamauchi et al. [137]	21	-	Japan	Plasma	Mutation	sequencing		Predictive
Liu et al. [138]	27	-	USA	Plasma	Methylation	Infinium HM450 array		Diagnostic
Takayama et al. [139]	85	-	Japan	Plasma	Concentration Mutation	dPCR	*KRAS*	Diagnostic Predictive
Toledo et al. [140]	1	-	Spain	Plasma	Whole Exome Sequencing	sequencing		Predicitve
Schou et al. [141]	123	-	Denmark	Plasma	Concentration	fluorescence		Diagnostic
Sclafani et al. [142]	51	-	Clinical Trial	Plasma	Mutation	ddPCR	*KRAS* *BRAF*	Diagnostic Predictive
Boysen et al. [143]	273	94	Denmark Norway Sweden	Plasma	Concentration	ddPCR DFA		Diagnostic
Myint et al. [144]	131	37	UK	Plasma Stool	Concentration Mutation	qPCR	*KRAS* *BRAF*	Diagnostic
Demuth et al. [145]	28	-	Denmark	Plasma	Mutation	ddPCR	*KRAS*	Prognostic
Rokni et al. [146]	50	-	Iran	Plasma	Methylation	High methylation resolution PCR	*BMP3*	Prognostic
Fu et al. [147]	98 CRC 101 adenomas 76 nCRC	253	China	Plasma	Methylation	MS-PCR	*SEPT9*	Prognostic
Molparia et al. [148]	24	25	USA	Plasma	Cnvs	sequencing		Diagnostic Prognostic
Gallardo-Gómez et al. [149]	20 CRC 20 adenomas	20	Spain	Serum	Methylation	microarray		Diagnostic
Nunes et al. [150]	72	103	Portugal	Plasma	Methylation	MS-PCR	*APC,* *FOXA1* *MGMT* *RARβ2* *RASSF1A* *SCGB3A1* *SEPT9* *SHOX2* *SOX17*	Prognostic
Song et al. [151]	150	-	China	Urine	Concentration	ddPCR		Predictive Prognostic
Yang et al. [152]	47	-	China	Plasma	Mutation	sequencing	*37 genes*	Diagnostic Prognostic
Suehiro et al. [153]	113	25	Japan	Serum	Methylation	ddPCR	*TWIST1*	Diagnostic
Sun et al. [154]	11	-	China	Plasma	Mutation	sequencing	*85 genes*	Prognostic
Thomsen et al. [155]	138	-	Denmark	Plasma	Concentration Mutation	ddPCR	*RAS/RAF*	Prognostic
Furuki et al. [156]	22	-	Japan	Serum	Mutation	sequencing	*TP53* *KRAS* *APC* *PIK3CA* *BRAF* *FBXW7* *NRAS*	Diagnostic Prognostic
Klein-Scory et al. [157]	3	-	Germany	Plasma	Mutation	BEAMing	*BRAF* *PIK3CA*	Predictive
Schøler et al. [158]	45	-	Denmark	Plasma	Concentration	WGS		Prognostic
Vandeputte et al. [159]	20	-	Belgium	Plasma	Concentration	ddPCR		Predictive

cfDNA: circulating cell-free DNA, ctDNA: circulating tumor DNA, CRC: colorectal cancer, ddPCR: droplet digital PCR; DFA: direct fluorescent assay; dPCR: digital PCR; MS-PCR: methylation specific PCR; qPCR: quantitative PCR; RT-PCR: real-time PCR; SOLID: Sequencing by Oligonucleotide Ligation and Detection; SSCP: single strand conformation PCR: WGS: whole genome sequencing.

## Abbreviations

ARMs	amplification refractory mutations system
BEAMing	bead emulsion amplification and magnetics
CAPP-Seq	cancer personalized profiling by deep sequencing
cSMART	circulating single molecule amplification and re-sequencing technology
cfDNA	circulating cell-free DNA
COLD-PCR	Co-amplification at lower denaturation temperature
CNV	copy number variations
CRC	colorectal cancer
CRT	chemoradiotherapy
CTC	circulating tumor cells
ctDNA	circulating tumor DNA
ddPCR	droplet digital PCR
DDR	DNA-damage response
dPCR	digital PCR
FISH	fluorescent in situ hybridization
MSI	microsatellite instability
MS-PCR	methylation specific PCR
NGS	next-generation sequencing
PNAs-LNA	peptide nucleic acid-locked nucleic acid
qPCR	quantitative PCR
RC	rectal cancer
RT-PCR	real-time PCR
Safe-Seq	safe sequencing system
SERS	surface-enhanced Raman spectroscopy
SNV	single nucleotide variation
SOLID	Sequencing by Oligonucleotide Ligation and Detection
SSCP	single strand conformation PCR
TAM-Seq	tagged-amplicon deep sequencing
UltraSEEK	high-throughput multiplex ultrasensitive mutation detection
WGS	whole genome sequencing
